# A Hybrid Assembly by Encapsulation of Human Cells within Mineralised Beads for Cell Therapy

**DOI:** 10.1371/journal.pone.0054683

**Published:** 2013-01-23

**Authors:** Philippe Dandoy, Christophe F. Meunier, Grégory Leroux, Virginie Voisin, Laetitia Giordano, Nathalie Caron, Carine Michiels, Bao-Lian Su

**Affiliations:** 1 Laboratory of Inorganic Materials Chemistry (CMI), The University of Namur (FUNDP), Namur, Belgium; 2 Laboratory of Molecular Physiology (URPhyM-NARILIS), The University of Namur (FUNDP), Namur, Belgium; 3 Laboratory of Biochemistry and Cellular Biology (URBC-NARILIS), The University of Namur (FUNDP), Namur, Belgium; Aristotle University of Thessaloniki, Greece

## Abstract

**Background:**

The design of new technologies for treatment of human disorders such as protein deficiencies is a complex and difficult task. Particularly, the construction of artificial organs, based on the immunoisolation of protein-secreting cells, requires the use of suitable materials which have to be biocompatible with the immunoisolated cells and avoid any inappropriate host response.

**Methodology/Principal Findings:**

This work investigates the *in vivo* behavior of mechanically resistant hybrid beads which can be considered as a model for artificial organ for cell therapy. This hybrid system was designed and fabricated *via* the encapsulation of living cells (HepG2) within alginate-silica composites. Two types of beads (alginate-silica hybrid (AS) or alginate/silica hybrid subsequently covered by an external layer of pure alginate (ASA)), with or without HepG2 cells, were implanted into several female Wistar rats. After four weeks, the potential inflammatory local response that might be due to the presence of materials was studied by histochemistry. The results showed that the performance of ASA beads was quite promising compared to AS beads, where less abnormal rat behaviour and less inflammatory cells in histological sections were observed in the case of ASA beads.

**Conclusions/Significance:**

The current study highlights that alginate-silica composite materials coated with an extra-alginate shell offer much promise in the development of robust implantation devices and artificial organs.

## Introduction

Living cell encapsulation currently attracts much interest owing to the new applications offered by this technology such as bioreactors, biocatalysis, biosensors or cell therapy [1]. In recent years, a variety of cell species, including yeasts [2], [3], bacteria [4], [5], plant cells [6]–[8] and animal cells, [9], [10] has been immobilised within inorganic-based materials. In the medical field, this technology is particularly promising to overcome the shortage of organ donors. In fact, the progress made in this specific domain could improve the compatibility between organisms and current encapsulating materials. For instance, in cell therapy, biocompatibility encompasses three major criteria: (1) the use of materials that are compatible with both the encapsulated cells and the human body (to target a graft for an artificial organ), (2) the development of synthesis methods that permit the *in-situ* construction of a matrix without damaging the cellular integrity and finally (3) the control of pore size in the host material, allowing nutrients and metabolites to permeate throughout the support [11].

Silica hydrogels have emerged as the prime materials to entrap living species since they can be synthesised through mild conditions (“*chimie douce*”) *via* the sol-gel process. The success of this technique is due to its flexibility in term of constructing materials with good mechanical and thermal stability, tuned pore size, as well as an adapted morphology. Nevertheless, the encapsulation of animal cells is a challenging task. In particular, immuno-isolation is a key factor to successfully develop cell therapy technologies where cells are protected against rejection by the immune system whilst allowing nutrients and metabolites to be evacuated. This protection can only be conferred by a biocompatible and semi-permeable membrane. Although previous works generally report a molecular weight cut-off (MWCO) around 150 kDa [12], [13], assigned to immunoglobulin G (IgG, the smallest antibody involved in the immune response), the pore size requirements for the membrane are still set as being between approximately 5 to 20 nm [14], [15]. Higher MWCOs could permit immune molecules to enter. Moreover, the materials should be sufficiently resistant with time to ensure long-term implantation of the graft. However silica materials have been reported as strong macrophage-attracting susbtances despite their overall advantages [16], [17]. Consequently, much research has been carried out using biopolymers such as polysaccharides to immobilise biological matter. For instance, sodium alginate crosslinked with calcium chloride has been found to be an excellent porous material for living cell encapsulation [18]. However, this ionotropic hydrogel presents the disadvantage of low mechanical strength and poor chemical durability [19]. Therefore, the properties of alginate materials need to be improved for efficient immuno-isolation. For these reasons, Carturan and Sakai have separately published two different methods for the fabrication of alginate-silica/alginate capsules [20]–[24]. In both cases, the procedure implies the preliminary formation of alginate beads encapsulating the cells before the deposition of an external silica shell, which is finally coated with Ca-alginate layer. In this way, the mechanical advantages of silica are exploited yet its drawbacks avoided. Nevertheless, in these materials, the silica component was only a thin layer formed at the biopolymer surface and not within the Ca-alginate hydrogel. However, it is well-known that thin porous silica films undergo a rapid dissolution under biological conditions (aqueous media, pH 7.4, 37°C) [25], [26] which compromises the long-term mechanical resistance of these capsules for clinical applications.

Very recently, we reported the encapsulation of HepG2 cells within resistant mineralised beads composed of two parts: an alginate-silica core and a Ca-alginate layer (ASA) [27] Human hepatocellular carcinoma cell line (HepG2) was chosen because they have similar size and morphology compared to β-cells. HepG2 can be thus used as a relevant cellular model to construct a prototype of bioartificial organs for the treatment of *Diabetes mellitus.* The primary results showed that entrapped cells can be kept alive for at least 6 weeks post-encapsulation. However, although various materials have been developed, few *in vivo* data are available to evaluate the benefits and drawbacks of silica hybrid materials. The aim of the present study was to investigate whether these silica-based materials induce an inflammatory response when implanted within rats for one month. More precisely, the relative *in vivo* biocompatibility of ASA materials was evaluated and compared to alginate-silica hybrid beads (AS) that are synthesised via a conventional synthesis method reported elsewhere [28]. To do so, muscle and subcutaneous tissues of Wistar rats, which surround the implants, were collected and analysed by histochemistry.

## Results and Discussion

### Bead Synthesis and Characterisation

The composition, porosity and robustness of materials play a major role to obtain implanted artificial organs with long-term efficiency. The use of polysaccharide hydrogels is commonplace, with alginate having been employed the most because of its recognised biocompatibility. Nevertheless, silica materials derived by the sol-gel process offer several advantages over biopolymers, such as negligible swelling in aqueous media, controllable porosity, chemical inertness, and hardness. In this work, two kinds of hybrid materials, which combined the advantages of alginate (e.g. biocompatibility) with those of silica (e.g. robustness), were synthesised under mild conditions (room temperature and atmospheric pressure).

**Figure 1:**
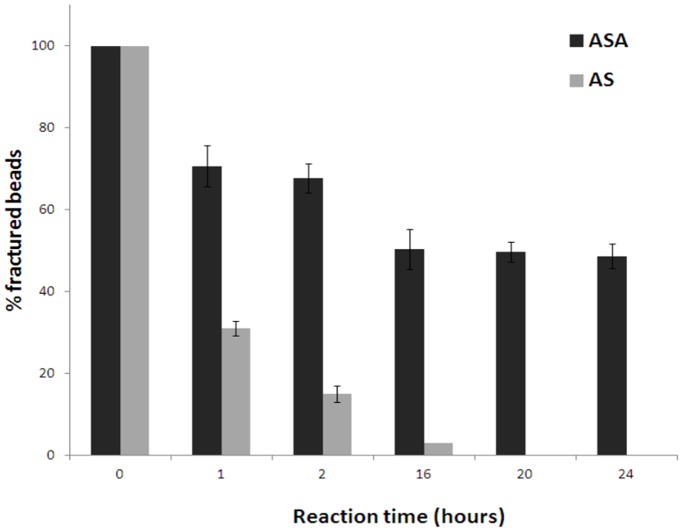
Evaluation of the mechanical resistance of beads. Comparison between AS and ASA beads.

**Figure 2:**
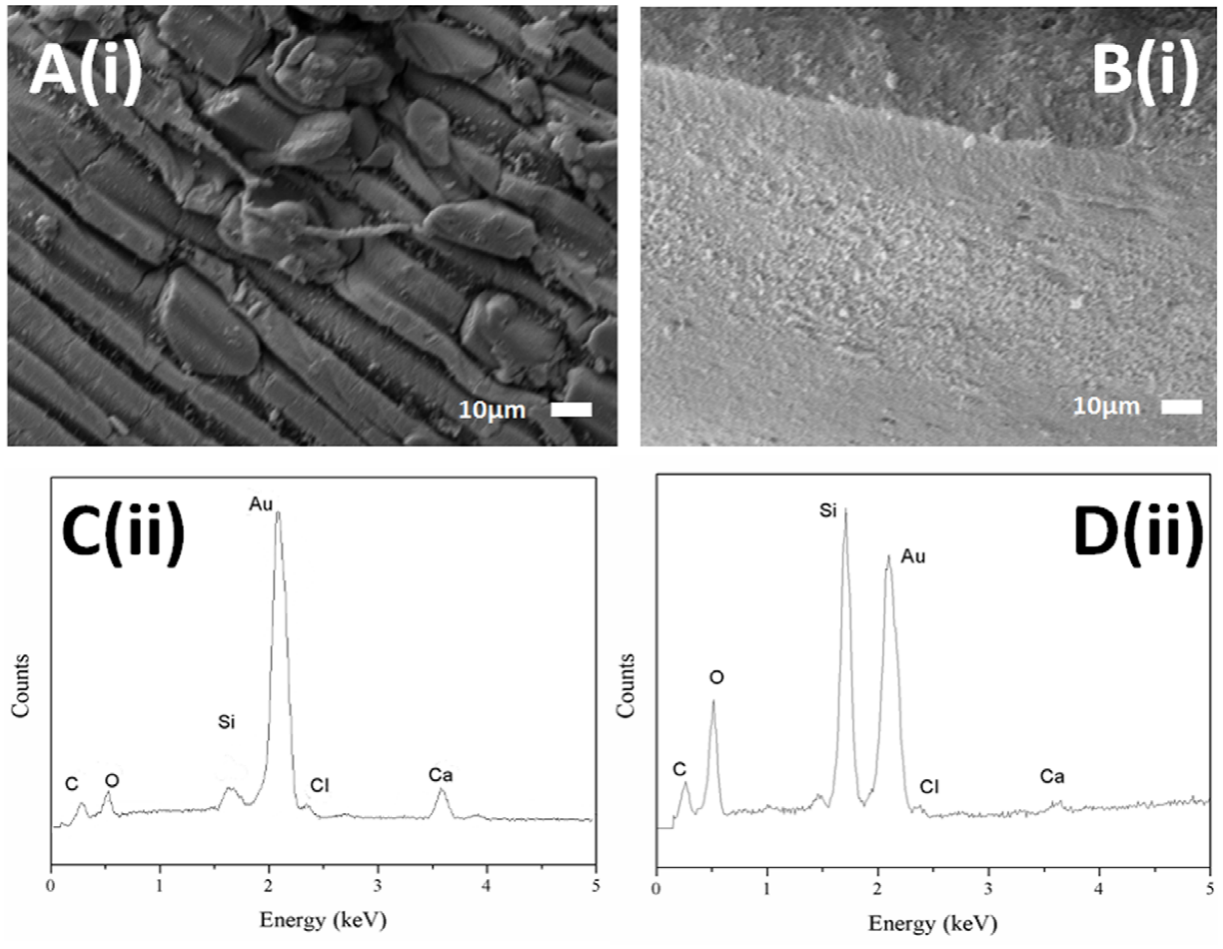
SEM micrographs (i) and EDX spectra (ii) of the beads. Characterisation of ASA bead (A, C) and AS bead (B, D).

Previous *in vitro* biocompatibility tests have shown an absence of a proinflammatory response from cells in direct contact with alginate or silica nanopowder [27]. These two materials were used to fabricate two types of mineralised beads: AS and ASA beads. The benefits brought from the mineralisation of the alginate capsule led to an increase in mechanical strength and a concomitant improvement in the control of the porosity of the encapsulating device. Before studying the *in vivo* behavior of materials, the hybrid beads were characterised by different techniques. The mechanical strength of the materials was firstly evaluated by stirring 100 beads at 60 rpm in 100 ml of a modified PBS buffer supplemented with EDTA (50 mM) and counting the fractured beads at selected intervals of time. This analysis medium was selected to mimic the major *in vivo* mechanism of degradation of alginate-based biomaterials due to the gradual exchange of calcium ions with sodium ions. As shown in Fig. 1, ASA beads are more resistant when compared to AS beads. SEM and EDX studies were undertaken to access the composition of the beads. As shown in Fig. 2(i), the external morphology of beads depended on the synthesis pathway. The AS bead surface was very smooth whereas the outer layer of ASA beads displayed a furrow-like morphology, characteristic of an alginate surface owing to the cross-linking contraction of the polysaccharide chains during the sample preparation. Additionally, a greater silicon to carbon ratio was detected in the case of AS beads as the EDX graphs (Fig. 2(ii)) show a higher intensity for the SiKα peak when compared to the ASA beads. These results suggest that the surface of AS beads is composed of a mix of Ca-alginate and silica whereas ASA beads are covered by an alginate layer.

As preliminary *in vitro* results suggested that these materials were biocompatible [27], *in vivo* experiments were performed to evaluate the inflammatory reaction within the surrounding tissues of these implanted beads.

### In vivo Experiments

In *vivo* experiments were conducted to compare the performance of AS beads and ASA beads by analysing the potential lesions of surrounding tissues of the beads four weeks after subcutaneous implantation within the back midline of female rats. Throughout the experiment, the body weight gain ratio was similar in both control and treated groups (Table 1), thereby indicating a good overall health and nutrition status of the rats.

**Table 1:** Weight gain ratio and use of astrexine during the *in vivo* experiment.

		Weight gain ratio	Use of astrexine
	Control	16%	1/3 animals (33%)
AS bead	Subgroup 1	27%	2/5 animals (40%)
	Subgroup 2	24%	4/5 animals (80%)
ASA bead	Subgroup 3	24%	1/5 animals (20%)
	Subgroup 4	18%	2/5 animals (40%)

Weight gain ratio of 2-month-old Wistar rats 1 month after implantation of AS and ASA beads; and use of astrexine for wound healing subsequent to the operation.

Some animals scratched the incision site and astrexine was used to promote healing of the inflamed wound. Astrexine facilitates the cicatrisation and relieves pain. More than half of the rats implanted with AS beads presented surgical site irritation and use of astrexine was required: in subgroup 2, only one animal did not present any sign of scratching. On the other hand, in the ASA beads group, only 3 out of 10 of the animals (subgroups 3 and 4) showed signs of scratching. This ratio was similar to the control group, thereby suggesting that inflammation was related to surgery rather than implanted ASA beads. These observations led to similar conclusions as those drawn by Arcangeli [16] who demonstrated that silica seemed to be an inappropriate material when it is in direct contact with living tissue. Additionally, table 1 highlights that the immuno-isolation of HepG2 cells entrapped within AS beads are quite poor as evidenced by the higher percentage of animals from the subgroup 2 showing signs of scratching around the surgical site. Conversely, ASA beads provide a better immuno-protection for HepG2 cells.

**Figure 3:**
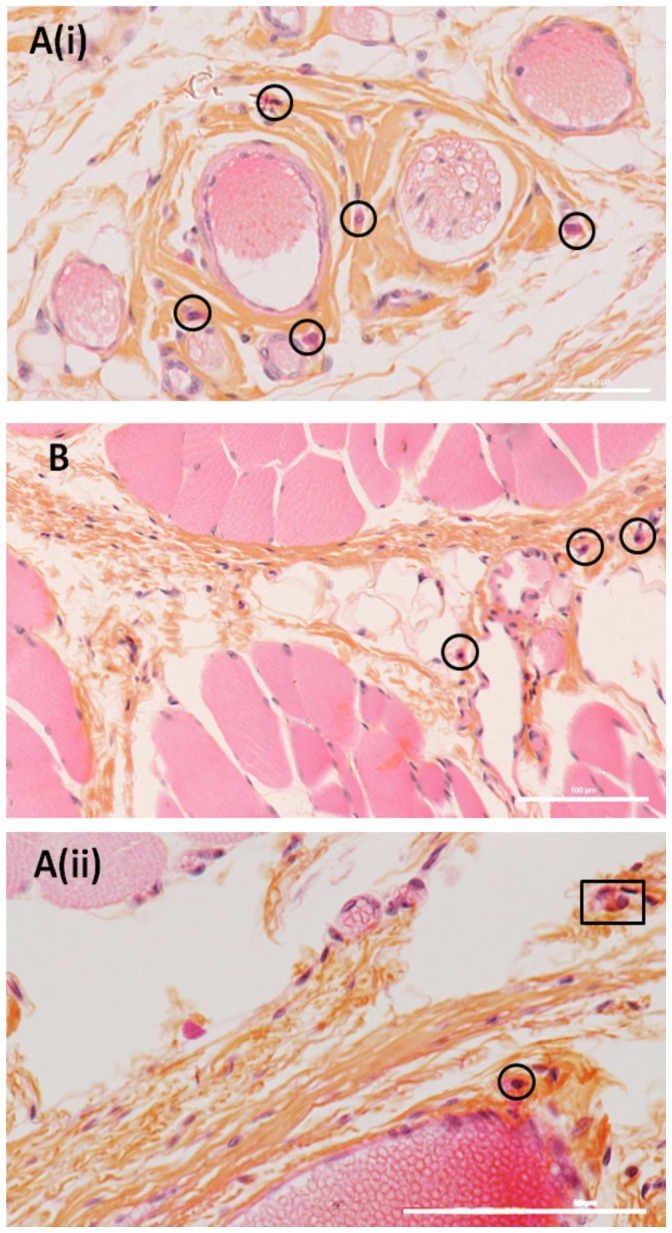
Histological sections of blood vessel (A) and muscle (B) of Wistar rats from the control group. Observation of histological sections from four AS beads without (1) and with (2) HepG2 cells implanted in their back midline for one month. High quantity of mast cells were found further away from the blood vessel, characteristic of potential alert. Mast cells are identified by circles and PML by squares. The scale bar corresponds to 100 µm.

**Figure 4:**
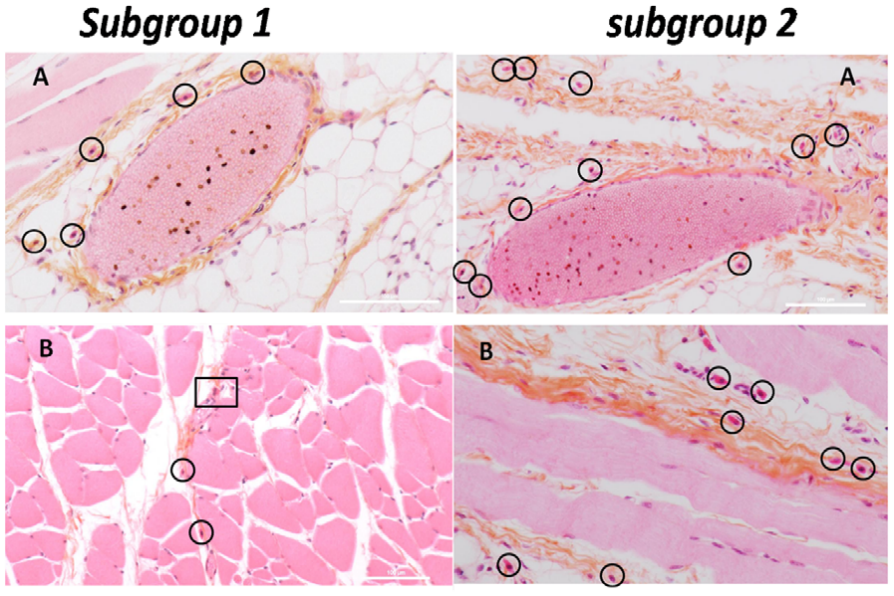
Histological sections of blood vessel (A) and muscle (B) of Wistar rats from subgroups 1 and 2. Observation of histological sections from four AS beads without HepG2 cells implanted in their back midline for one month. High quantity of mast cells were found further away from the blood vessel, characteristic of potential alert. Mast cells are identified by circles and PML by squares. The scale bar corresponds to 100 µm.

**Figure 5:**
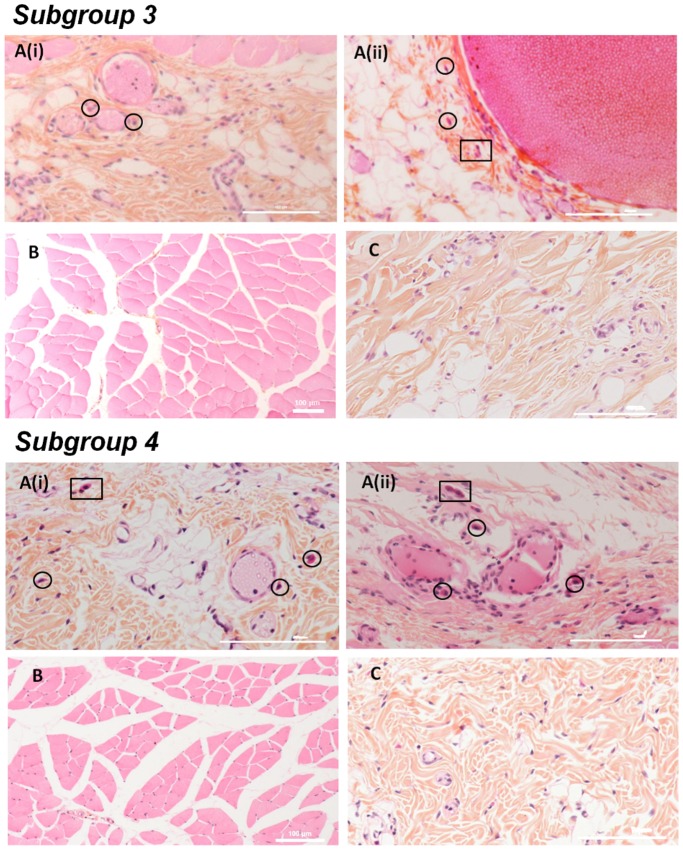
Histological sections of blood vessel (A), muscle (B) and collagen (C) of Wistar rats from subgroups 3 and 4. Observation of histological sections from four ASA beads without (3) and with (4) HepG2 cells implanted in their dorsal midline for one month. Mast cells are identified by circles and PML by squares. The scale bar corresponds to 100 µm.

**Figure 6:**
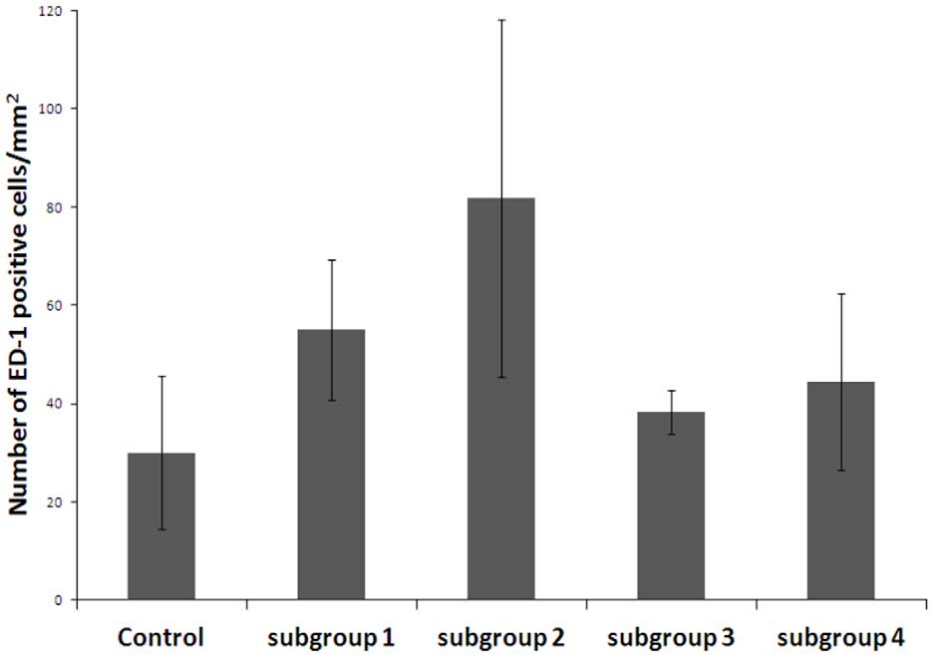
Macrophages quantification localized in tissues surrounding the implants (ED-1 positive cells). Data are means ± SD of four replicates.

Histological sections of the subcutaneous and muscle tissues surrounding the implanted beads were performed to look for the presence of mast cells and polymorphonuclear leukocytes (PML). These cells are found near blood vessels in normal tissues, as sentinel cells. These sentinels are identifiable owing to their purple nucleus surrounded by a strong-red staining, especially for PML which can display various shapes, usually bi- or tri-lobed. Pictures from the control group in Fig. 3 confirmed the presence of these resident cells in all the rats. Figs. 4 to 5 highlight sections for all four subgroups of treated rats. No necrosis or lesions in the tissue were observed in any sample. PML and mast cells were observed in subgroups 1 to 4 (AS and ASA beads) but their presence was not high enough to conclude that inflammation or rejection was occurring. However, in comparison to the control group, more indicators of rejection were observed in subgroups 1 and 2 (AS beads), and to a lesser extent in subgroups 3 and 4 (ASA beads, Fig. 6). Usually found close to blood vessels, mast cells were detected in higher abundance much further away in the AS subgroups (Fig. 4A). They were also present around muscles (Fig. 4B). In some cases, mast cells and PML were even found between the muscles (Fig. 4B).

**Figure 7:**
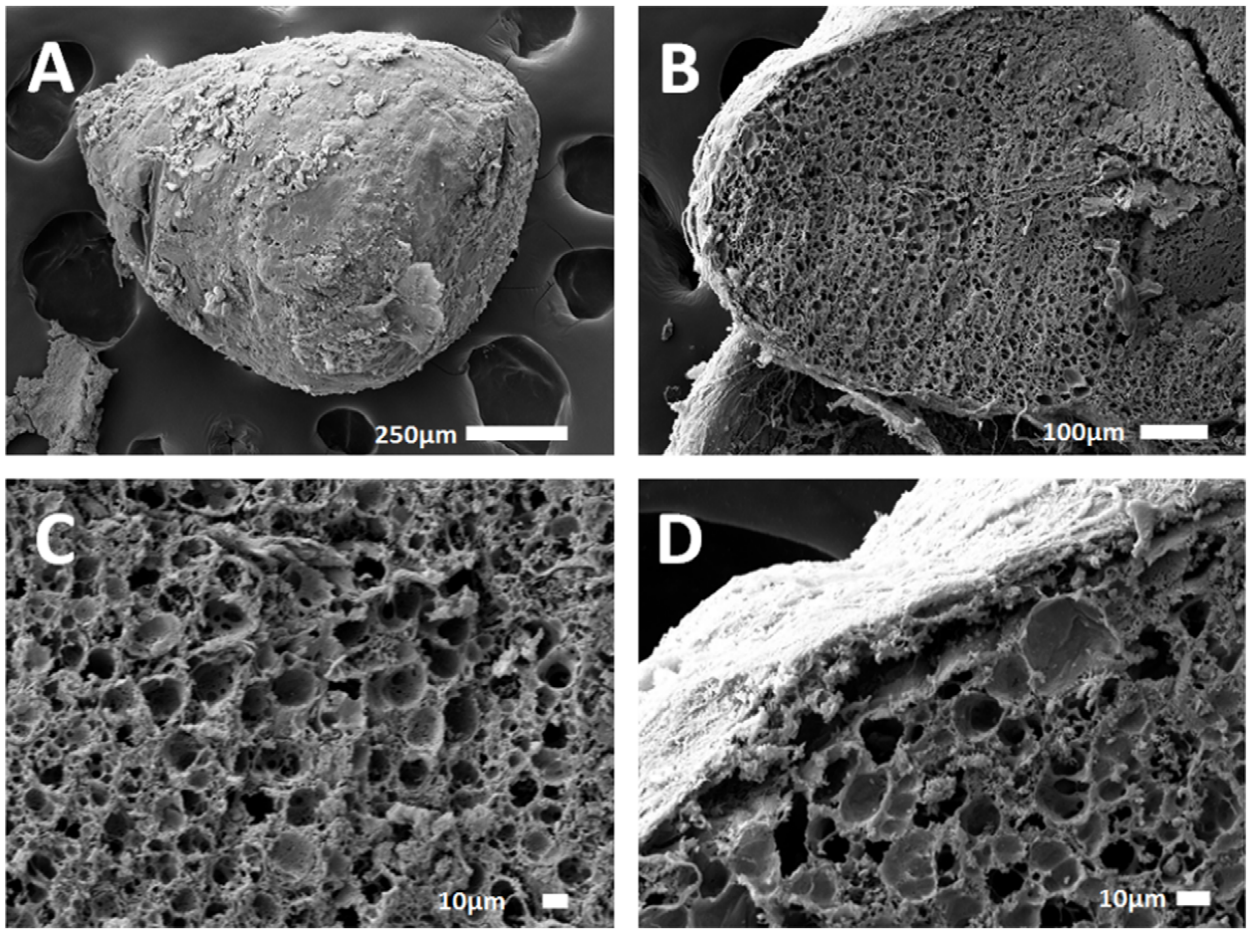
SEM micrographs from subgroup 4. ASA bead with encapsulated HepG2 cells observed one-month post-implantation *in vivo*.

The quantity of mast cells was much higher near the blood vessel in subgroup 2 than the quantity found around one blood vessel of the control group. For instance, Fig. 4A (subgroup 2) displays about two times more mast cells than Fig. 3A (control). The inflammation factors were thus more numerous for subgroup 2, where HepG2 cells were encapsulated inside the AS beads. Histological observations of subgroup 3 (ASA beads, no cells) suggested that the tissues were less inflamed (Fig. 5). Mast cells and PML were located close to the blood vessel. Either in collagen or in muscles, blood vessels did not repatriate many leukocytes. In subgroup 4 (ASA beads, entrapped HepG2 cells), the mast cells were mainly situated close to the vessels and not deep into the collagen or connective tissue (Fig. 5). Behaviour assessments and histological observations of the tissues of rats used in these *in vivo* experiments suggest that the ASA beads appeared to have a more pronounced biocompatibility than AS beads. Furthermore, ASA beads were still intact one month post-implantation, as shown by the SEM micrographs from subgroup 4 (Fig. 7). The furrow-like external shape of the ASA bead seemed to be lost. As the connective tissue encircled the beads very well, the external crust of the beads was probably took off during the preparation of these samples. The second hypothesis is that in the body fluids, monovalent ions could erode the alginate crust to get this final smooth surface. A cross-section of ASA beads (Figs. 7B–D) reveals the presence of many pores that were initially filled with HepG2 cells which were pulled out during the drying process prior to observations. The well-defined difference of morphology between the outer layer and the inside part demonstrate that the alginate crust was preserved *in vivo* (Fig. 7D). In the case of AS beads, no electron microscope micrographs could be obtained as the connective tissue around them was too tightened to isolate the beads alone during preparation for observation.

### Conclusions

*In vivo* tests on new hybrid materials were carried out in order to evaluate their biocompatibility when they are implanted within living organisms. Two kinds of sol-gel synthesised materials enclosing living HepG2 cells were prepared: alginate-silica beads (AS) and alginate/silica hybrid subsequently covered by an external layer of pure alginate (ASA). Consequently, AS beads are essentially distinguishable from ASA beads by their surface chemistry. The results show that the mineralised ASA beads covered by an external alginate crust are promising hybrid materials since fewer animals displayed localised inflammatory, compared to conventional alginate-silica composites (AS beads). As integrity of ASA beads was preserved during this *in vivo* study, we are very optimistic about their efficiency for further clinical applications.

## Materials and Methods

### Materials

Calcium chloride (dihydrate, 99%), sodium alginate powder (sodium salt from brown algae), ethylene glycol tetraacetic acid (EGTA, 99%), hydrochloric acid (HCl, 37%), chloroform (99%), butanol (99%) were provided by Sigma-Aldrich; and sodium silicate (assay 25.5–28.5% SiO_2_) by Merck. The HepG2 cell line was purchased from the American Type Culture Collection (ATCC No HB-8065). The Dubelcco’s Modified Eagle Medium (DMEM) and the foetal bovine serum were obtained from Invitrogen (Carsbald, USA)_._ Before used, commercial sodium alginate powder was purified according to the method published by de Vos [29].

### HepG2 Cells Culture Conditions

Encapsulation was carried out using human hepatoma HepG2 cells (Hepatocellular Liver Carcinomia Cell line) cultivated in 75 cm^2^ polystyrene flasks (Costar, Lowell, USA) with 18 ml DMEM medium and 10% foetal bovine serum, and incubated at 37°C in a humid atmosphere of 5% CO_2_.

### Encapsulation Process

Prior to encapsulation, cells were first washed with a sterile phosphate buffer solution (PBS), trypsinised using trypsin/EDTA and centrifuged at 1000 rpm for 4 min. The supernatant was discarded and fresh DMEM medium+serum were added to resuspend the HepG2 cells.

#### Type AS

The first route of encapsulation used the method described by J. Livage and coworkers with some modifications [28] The alginate-silica beads were obtained by mixing cells, in suspension within their buffered culture medium (pH = 7.4), with purified Na-alginate (1.6% wt). A sodium silicate solution (1.5 M) was then added (1∶6 vol.) and the mixture dropped into a calcium chloride solution (110 mM) using a syringe with a 24G-needle in order to crosslink the alginate and silica to form the AS beads. The final cell density was 1.5 10^6^ cells ml^−1^ and the diameter of AS beads was 2.85(±0.01) mm.

#### Type ASA

Secondly, the alginate-silica/alginate beads were synthesised according to the 3-step procedure previously reported [27]. The cells suspended in their buffered culture medium (1.5 10^6^ cells ml^−1^) were first encapsulated within Ca-alginate beads (1.6% wt), by dropping a cell culture/Na-alginate mix into a CaCl_2_ solution (110 mM) using a syringe with a 24G-needle. The alginate capsules were then placed in a sodium silicate solution (1.5 M) for a short period of time (30 s). The mineralised beads were then collected and an external Ca-alginate crust was formed by re-suspending the beads in 1.5% sodium alginate solution for 3 min. Finally, the hybrid materials were dipped into a CaCl_2_ solution (110 mM) in order to crosslink the outer sodium alginate layer. The diameter of ASA beads was 2.55(±0.09) mm.

Using an optical microscope, the efficiency of entrapment was checked by measuring the numbers of HepG2 cells that could grow out of the beads. In both cases, no cell was detected.

### Characterisation Techniques

#### Ethics statement

The present study is conformed to the National Institutes of Health Guide for the Care and Use of Laboratory Animals and the protocol was approved by the Committee on the Ethics of Animal Experiments of the University of Namur (Permit Number: FUNDP AR 10/136).

#### Animals

The experiments were performed on 8 weeks old female Wistar rats bred in our animal facility under controlled environmental conditions. The animals had free access to water and were fed with a standard rat chow diet.

#### Surgical procedure

Prior implantation step, cell viability was assessed and 90% of cells, homogenously dispersed within the AS or ASA beads, were still alive. The rats were anesthetised with a mix of ketamine/xylazine (2 ml/kg i.p. of a 2% ketamine/0.3% xylazine isotonic saline solution) and placed on a heated table to maintain body temperature between 37 and 38°C. A skin incision was performed along the back midline between the shoulder blades. Two beads were then implanted subcutaneously on each side of the surgical site. The incision was closed using Ethicon Vicryl suture 4–0 thread and the rats were allowed to recover. Throughout the four weeks experiment, daily observations were made related to the welfare and behaviour of the animals. This assessment consisted to manipulate the animals, observe their rodent habits, detect fear, anxiety, stress, discomfort and identify wound or trace of itching around the surgical site. Treatment with astrexine (chlorhexidin hydrochlorid, Pierre Fabre, France) applied on the skin surface was only performed when signs of inflammation were detected by daily examination. Four weeks later, the rats were submitted to euthanasia (200 mg/kg Nembutal i.p. followed by exsanguination) to allow collection of the beads as well as sampling of the surrounding tissue, in order to observe potential local inflammation.

#### Experimental groups

Three groups were considered:

- Control group (n = 3): the rats were only submitted to anaesthesia, surgery and recovery;
- AS bead implants (n = 10) and ASA bead implants (n = 10) groups. These two groups were subdivided in two subgroups, rats were randomly implanted with either blank beads containing no cells (subgroup 1 and 3, n = 5 each) or with beads containing HepG2 cells to evaluate the immuno-isolation (subgroup 2 and 4, n = 5 each).

### Histological Staining

Muscle and subcutaneous tissues collected around the beads were fixed in Duboscq-Brazil fluid, embedded in paraffin and cut into 6 µm thick sections, for histological staining purposes. To allow histological examination, a trichromic stain was employed using the HES protocol (Haematoxylin – Eosin– Safranin) : the slides were submitted to successive baths of toluene, methanol, water, haematoxylin, water, hydrochloric acid+ethanol, water, eosin, water, ethanol, isopropanol, safranin, isopropanol and toluene using a continuous linear stainer COT 20 (Medite Inc.). Each bath lasted 150 s with a dripping step of 10 s between baths. Coverslips were attached with DPX (a mixture of Distyrene, a Plasticizer (tricresyl phosphate), and Xylene) and the slides were observed using a Multizoom AZ100 microscope (Nikon) with a DS-Ri1 camera in bright-field mode.

Using immunohistochemistry, macrophages were identified using ED-1 antibodies. Briefly, deparaffinized sections were pretreated with a citrate buffer and endogenous peroxidase activity was eliminated by a treatment with hydrogen peroxide for 5 minutes. Sections were then successively incubated with avidin, biotin and casein proteins to block nonspecific antibody binding sites. Primary antibody (ED-1, 1∶50) was incubated during 30 minutes. Thereafter, sections were incubated with biotinylated anti-mouse immunoglobulins (1∶50). Finally, the avidin-biotin method was employed to stain tissue sections [30] The numbers of ED-1-positive cells were quantified. For each tissue section, 4 representative fields were chosen, and the average number of macrophages was calculated and expressed as number of cells per mm^2^.
